# Development of an Auxiliary Platform (Mentali) for the Primary Screening of Anxiety and Depression in Young Adults

**DOI:** 10.3390/ijerph192114033

**Published:** 2022-10-28

**Authors:** Jorge Alfonso Solis-Galvan, Sodel Vazquez-Reyes, Idalia Garza-Veloz, Perla Velasco-Elizondo, Alejandro Mauricio-Gonzalez, Margarita de la Luz Martinez-Fierro

**Affiliations:** 1 Molecular Medicine Laboratory, Academic Unit of Human Medicine and Health Sciences, Universidad Autonoma de Zacatecas, Carretera Zacatecas-Guadalajara Km.6. Ejido la Escondida, Zacatecas 98160, Mexico; 2 Academic Unit of Electrical Engineering, Universidad Autonoma de Zacatecas, Carretera Zacatecas-Guadalajara Km.6. Ejido la Escondida, Zacatecas 98160, Mexico

**Keywords:** mental health, anxiety, depression, mobile application, web application, mobile, app

## Abstract

The current COVID-19 pandemic has completely changed people’s daily routines. This has had a big impact on mental health. In Mexico, medical school authorities are interested in understanding the mental health status of the student population to be able to provide support to students who may need help from a mental health specialist. The aim of this study was to develop a platform comprised of a mobile and web application called Mentali, to be used as an auxiliary tool for the detection of conditions such as anxiety and depression, as well as variations in mood, by analysis of the results of validated inventories. Following the Scrum software development methodology, Python, Dart and PHP programming languages were used for development of the application. This platform was used prospectively with 155 first year students taking part in the human medicine program. After 22 weeks, Mentali enabled the identification of 40 users with positive primary screening for anxiety and/or depression (45% for anxiety, 32.5% for both anxiety and depression, and 22.5% for altered mood). These students were contacted and referred to a psychologist; however, only 26 (65%) accepted psychological support. For all of these students a mental health disorder was confirmed. The results support the use of Mentali for the primary screening of anxiety and depression in young adults, including medical students.

## 1. Introduction

The World Health Organization predicts that by 2030 mental illness will represent the main burden of disease worldwide. Mental health conditions have a significant impact on people, and some, such as depression, have the greatest impacts on young people [1,2]. For example, in Mexico, the results of the National Survey of Psychiatric Epidemiology (ENEP acronym in Spanish) indicated that approximately one in five individuals will experience at least one mental disorder at some point in their life [3]. There are a variety of factors that may cause these conditions in people, including work, economic, school and family factors [4]. In addition, the coronavirus disease 2019 (COVID-19) pandemic has completely changed people’s daily routines, for example, from working in an office to working from home, from studying in a classroom to studying through digital platforms without contact with teachers and classmates, and people often having to stay at home all day. This situation might have a significant effect on the mental stability of society [5]. As a result, there has been increasing interest in developing solutions to facilitate support by mental health professionals.

University students represented a vulnerable population even before the pandemic [6]. This is because students’ psychological state can be influenced by the university environment, which can become an area of mental health risk due to difficulties students face in meeting school requirements and in dealing with highly demanding and stressful situations. In this project, we focus on the area of health sciences, specifically, students pursuing a bachelor’s degree in human medicine. Such students are subject to very high workloads and complicated schedules and work in a very competitive environment, which are factors that may be associated with symptoms of depression and physical harm [7].

Previously, at the Universidad Autonoma de Zacatecas, screening for anxiety and/or depression using psychometric tests, such as the Beck anxiety and depression inventories [8,9] were administered on paper; however, this approach has the disadvantage of delay in provision of results when the screening is undertaken in large populations. The validity of the psychometric tests themselves is not in question; however, since the concern is with problems related to mental health, it is essential that the patient who requires it receives attention immediately.

Given the impact that anxiety and depression can have on the well-being of students, the importance of developing the platform was recognized. The platform seeks to solve the problems faced by university authorities in obtaining information related to the mental health of the student population. By obtaining the results of the inventories more quickly, it will be possible to identify those students who require psychological attention, who can then be directed towards a relevant specialist. This should prevent further negative consequences.

The use of mobile phones has made it possible for mobile-based mental health interventions to be adopted as an increasingly popular approach to addressing barriers to accessing mental health services [10]. Currently, university students often engage in prolonged use of mobile phones. As a key aspect of the platform developed, this led us to consider inclusion of a mobile app to facilitate students being able to respond to the anxiety and depression inventories.

We conducted a systematic literature review with the aim of identifying mobile applications that have been developed in the field of mental health. According to the reviewed articles, currently, there are many mobile applications (more than 100) for which the main objective is to help improve mental health. Most of these applications focus primarily on treating conditions such as depression, anxiety, and stress. However, a significant proportion of these applications do not meet the needs of their potential users, which causes them to be underused [11]. The review enabled us to identify possible challenges in the development of mobile applications in the field of mental health, as well as possible strategies to overcome these challenges (for more details of the systematic review, please see Solis-Galvan, J.A. et al., 2020) [11].

Based on the above, the aim of this study was to develop a platform comprised of a mobile and web application called Mentali, to be used as an auxiliary tool for the detection of anxiety and depression based on analysis of the results of validated inventories and including assessment of a user’s mood. This article presents the development process of the Mentali platform and the results obtained from a pilot study.

## 2. Materials and Methods

**Figure 1:**
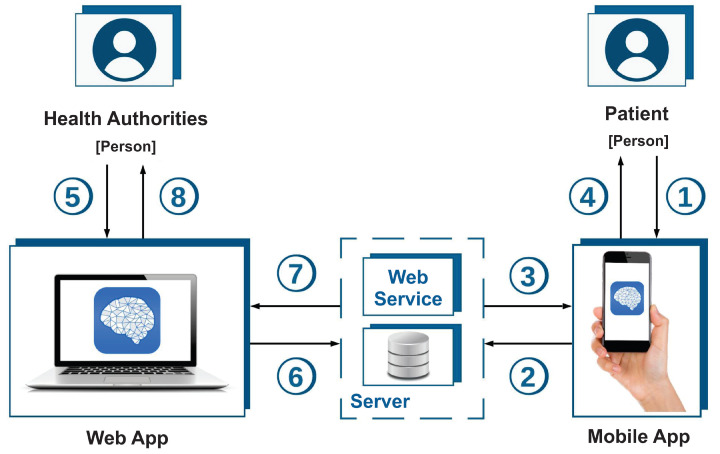
Platform diagram. The mobile application and the web application interact via a web service and a database to create a single platform. When using the mobile application, the student can complete the auxiliary inventories for the diagnosis of anxiety and depression, as well as recording their mood throughout the day. The mobile app stores the information in a database, using the web service. When required, the mobile app extracts information from the student’s mood logs from the database, so that the student can visualize graphs and diagrams with information about their mood logs. A specialist/administrator can also generate reports based on the results of the inventories and mood of students via the web application, which transfers information from the inventories and state of mind records to the database. The information is extracted from the database according to the criteria indicated by the specialist, who, in addition, can, by using the web application, visualize information derived from the inventories and the mood of the students as a basis for decision-making.

The platform described in this research was developed by the Molecular Medicine Laboratory at the Universidad Autónoma de Zacatecas in Mexico and consists of two software systems, as shown in Figure 1. To develop the platform, we used the Scrum software development methodology [12]. This method was used because the features of the software developed demanded constant communication with users as well as feedback from different versions. The platform included a mobile app called Mentali, which was used by the medical students and enables users to respond to anxiety and depression inventories. For this platform, the Beck Anxiety Inventory and the Beck Depression Inventory were selected [8,9]. In addition to providing answers to the depression and anxiety inventories, the students also recorded their mood. This involved selecting one of the mood options presented (Excellent, Good, Regular, Bad, and Terrible ) and answering the question: ’What activity are you performing right now?’. In this way, users are able to keep track of their mood and to review their behavior. The second element of the platform is the web application which is used by administrators with the purpose of identifying those students who might require the attention of a psychologist. In addition, specialists/administrators using the platform can review information from the inventories and mood measures in different ways, for example, using tables, graphs, or even word clouds. Both of the applications comprising the platform interact through a web service and a database which are hosted on a server.

**Figure 2:**
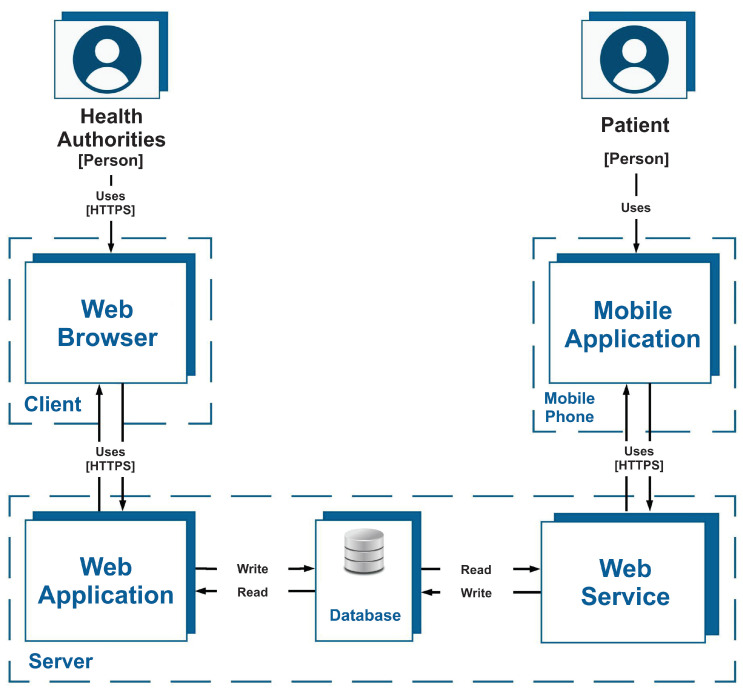
Static view of the platform. The deployment of the mobile app took place in the three main mobile app stores. Deployment of the web application was performed on the web server using Docker.

From the above diagram, Figure 1, a static view of the platform was developed. This view is presented in Figure 2, which illustrates how each of the components of the platform are deployed. The mobile app is installed on the student’s smartphone which uses a web service that is hosted on a server along with the database and the web application. The web service processes the information and stores it in the database, to which the web application also has access in order to process requests for the generation of reports and graphs. Administrators can access the web application through a web browser installed on their personal computer.

### 2.1. Mobile Application

Due to the importance of the mobile application within the platform and according to the needs identified, it was developed first. The methodology used for development of the software involved incremental development—the essential features were implemented in the first iterations and then the complementary features were developed.

#### 2.1.1. Architecture

**Figure 3:**
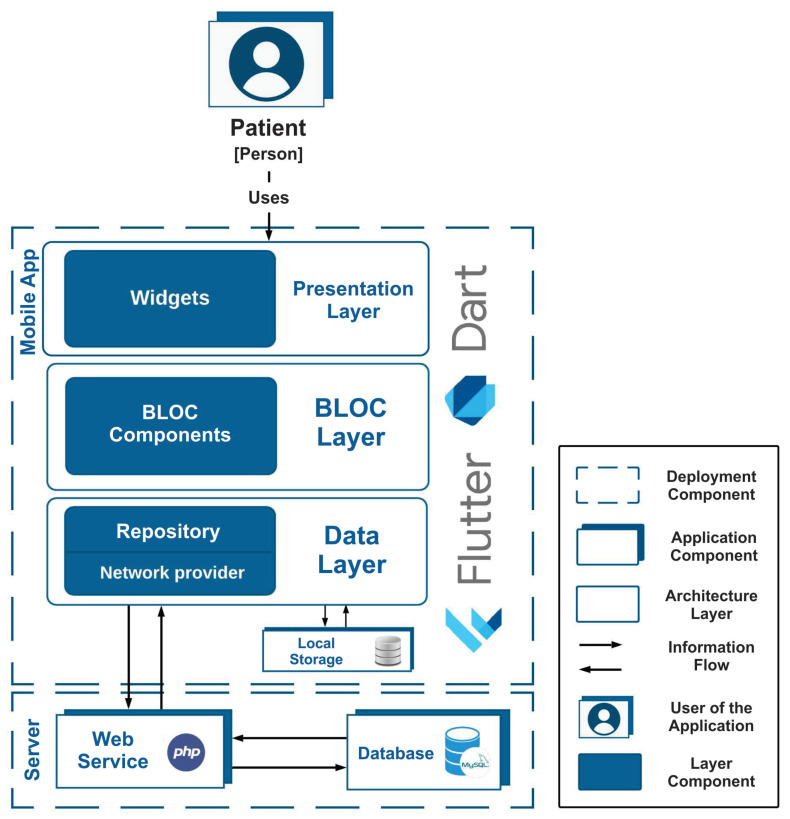
Static and physical view of the mobile app.

The general architecture of the application was developed as the first activity in the design phase of the application. Figure 3 represents a static and physical view of the architecture.

Within the architecture development process, several design decisions were made. It was decided to use the reference architecture for mobile devices [13]. In this architecture, there are two main components, which represent the deployment components of the mobile application and the server hosting a web service and the database. Within the mobile application component, the architectural pattern is used to structure the application modules and access the web service that enables linking to the database.

The pattern used for the web application is called the BLOC pattern and requires that the application is formed of four general components which are distributed in three layers according to its functionality. The three layers are the presentation layer, the BLOC layer and the data layer. These layers are represented in the diagram by white rectangles with blue edges and rounded corners.

For development of the mobile application, the decision was made to use the Dart [14] programming language with the Flutter [15] development framework. One of the main characteristics of Flutter that motivated this choice is the speed with which it is possible to develop mobile applications. In addition, Flutter includes components that enable the generation of expressive and flexible user interfaces, a valuable feature because one of the key challenges in the development of an application is to ensure its usability. Finally, Flutter allows apps to be produced for iOS and Android platforms from the same source code, which requires reduced development time without sacrificing performance. In Figure 3, the technology selection process for each component of the mobile app is shown.

The application uses a web service developed in PHP which performs the processing of information, as well as enabling the storage and extraction of data in the database. The service is hosted on a server provided by Amazon Web Services. The decision was made to host the service at Amazon because this company provides safe, durable and scalable services, which are subject to recognized certification and audit procedures.

#### 2.1.2. Database Diagram

**Figure 4:**
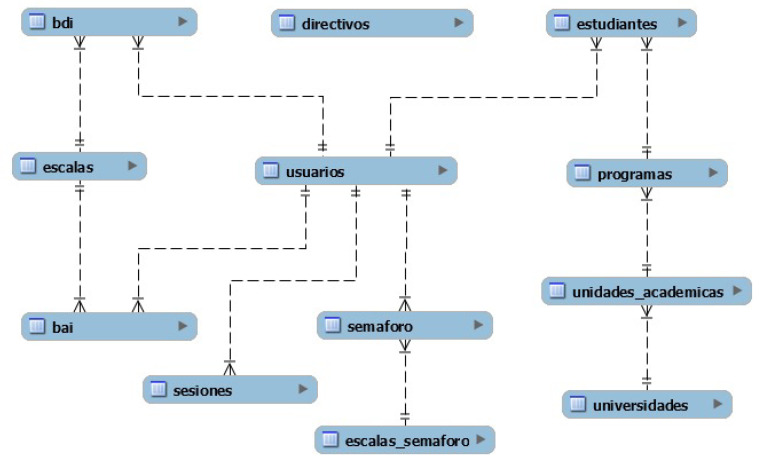
Mobile app database diagram. The diagram satisfies the requirements of the mobile application; feedback obtained in the first iteration was incorporated.

As a next step, the database diagram was developed. In each of the iterations the tables that would be necessary for the storage of the information and to satisfy each of the requirements were formed. In the diagram for the first iteration, the entities needed to meet the requirements of user account creation, login and inventory registration were recorded. For the second iteration, the necessary entities to satisfy the requirements for registration of mood were formed and the entities to store the information for the academic programs, academic units and universities as part of the feedback from the first iteration were added. Figure 4 shows the database diagram.

#### 2.1.3. Design

**Figure 5:**
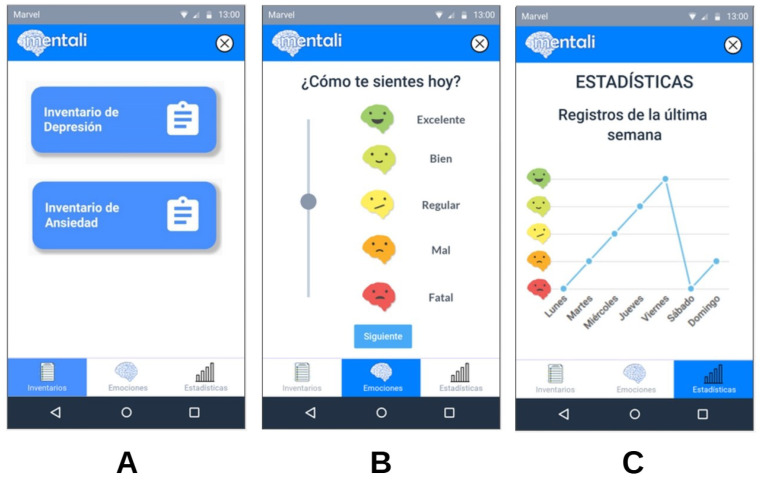
(**A**–**C**) Mobile app mockups.

As part of the design phase, "mockups" of the mobile app screens were developed. For the first iteration, user interface designs were generated to address the initial requirements related to the anxiety and depression inventories (see Figure 5A). For the second iteration, modifications were made in the design of the screens according to feedback from users. This iteration addressed the recording of mood and the visualization of statistics for these records (see Figure 5B,C).

### 2.2. Web Application

For the platform to meet the identified needs, it was necessary to develop a web application. This application aims to offer a simple way to generate reports and visualize statistics about the data generated through the use of the mobile application by users. Because user data are considered sensitive, only specialists/administrators have full access to this application.

#### 2.2.1. Architecture

The first design decision made for the web application was the implementation of an MVC architecture. A pattern widely used in web systems allowing separation of components according to their main purpose was used.

**Figure 6:**
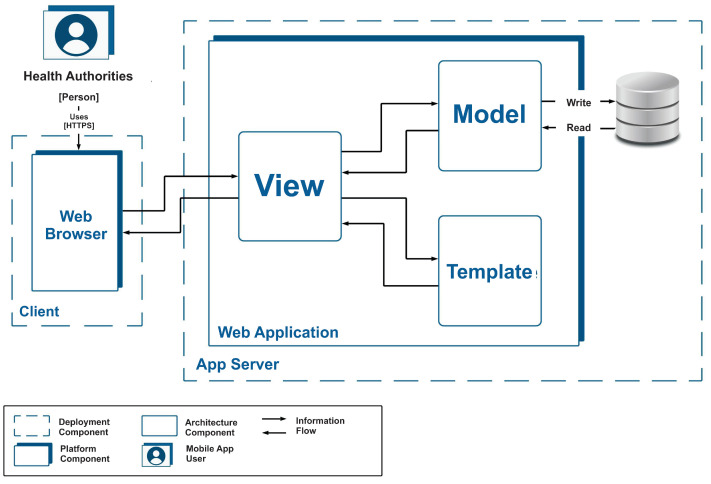
Static and physical view of the web application.

As a second design decision, the use of the Django [16] development framework, which is used as the Python [17] programming language, was considered. This framework supports the MVC pattern but with some small differences in the definition of the main components. Instead of dividing the components into model, view and controller, the Django framework uses model, view and template. In the MVT pattern, the template represents the views that are presented to the user and the view represents the controller that handles user interaction. Figure 6 shows a static and physical view representing the web application.

In the view, it is possible to see that the entire application is deployed on an application server, where the database where the information for the operation of both applications is stored and extracted is also located. Within the limits of the web application the main components of the architecture, the view, the model and the template, can be seen. When the user makes a request from the browser, this request reaches the view component, which decides whether it is necessary to use the template to display the user interface or the model to perform storage or extraction of information from the database.

#### 2.2.2. Database Diagram

**Figure 7:**
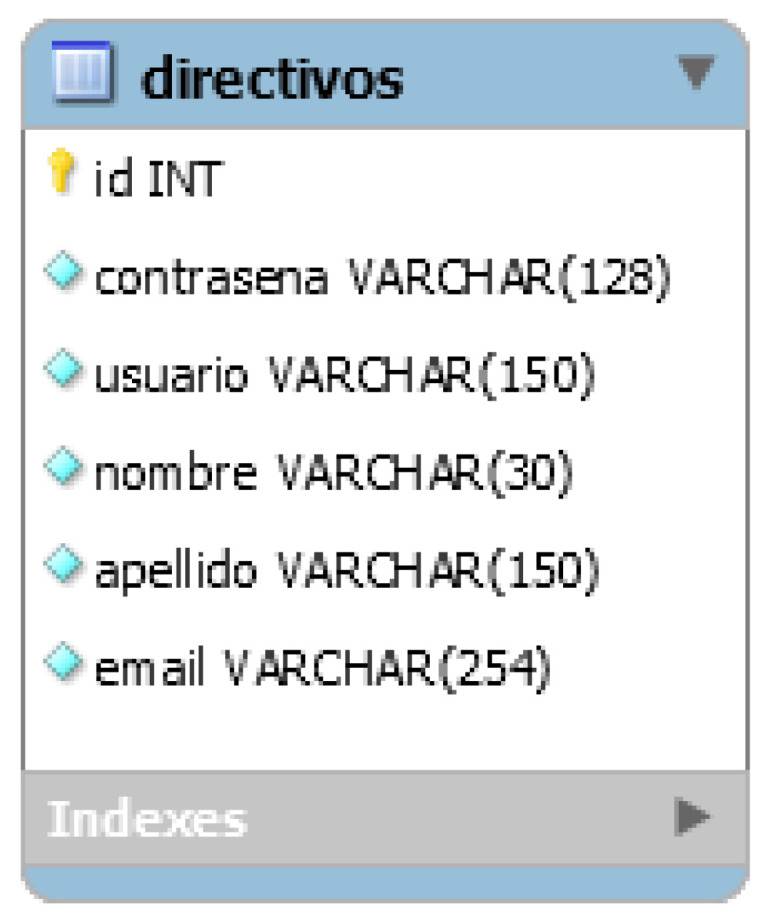
Web application database diagram. Since the mobile app web service and web app share the database, only one additional table was added for the web app.

According to the general architecture of the platform shown above, the mobile application and the web application perform storage and extraction of the information from the same database hosted on an external server. For this reason, the database diagram of the web application is the same as that of the mobile application, only the Directivos table is added, which is shown in Figure 7.

#### 2.2.3. Design

As with the mobile app, the mockups of the main screens were designed for the web app. For the first iteration, user interface designs were generated to address the initial requirements of the web application. These requirements are related to the creation of user accounts for specialists to login to the system.

For the second iteration, a general screen design was developed to cover user stories for the visualization of inventory records and the generation of the reports of these records. In the third iteration, the designs of the visualization of the mood registers and the production of the reports of these registers were generated.

## 3. Results

In this section, we present the results of the overall platform development process, namely the mobile application and the web application. The development, testing and deployment phases are presented in each application section.

### 3.1. Mobile Application

As mentioned above, the mobile application is a fundamental part of the Mentali platform as it generates the necessary information to meet the main objectives of the project.

#### 3.1.1. Participatory Design Workshop

As the main strategy to avoid usability problems in the mobile app, a participatory design workshop was implemented attended by potential users. In this workshop, students of the Academic Unit of Medical Sciences at the Universidad Autónoma de Zacatecas (UAZ), in Zacatecas Mexico, were involved as potential users of the application. This was one of the activities that allowed us to obtain more feedback from users. In this workshop, activities were carried out to determine the preferences of users from different perspectives. The workshop allowed us to identify interesting characteristics of users. First, users reported a high frequency of mobile phone usage during the day, which helped support our proposal for mobile app development. According to the activities carried out, users showed interest in their mental health and expressed willingness to use a mobile application that could help them to take care of their mental health. However, they expressed concerns about the privacy of their personal data. Finally, the most frequent recommendations obtained from users were related to the availability of an application 24 h a day, seven days a week, which was visually attractive and supported by mental health specialists.

#### 3.1.2. Development

**Figure 8:**
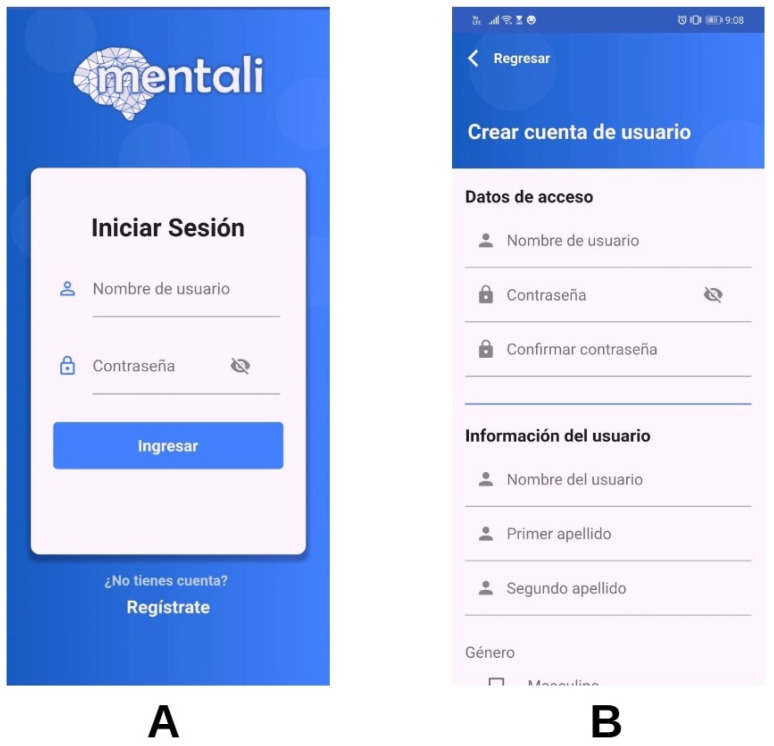
Login (**A**) and new account (**B**) screens.

As a result of this phase, screenshots of the mobile app modules were displayed. As part of the development of the mobile application, the user and inventory register modules were developed in the first iteration. With implementation of the user module, users can create a user account and login to the application. Once logged in, users can interact with all the available features. In Figure 8A,B screenshots of this module are shown.

**Figure 9:**
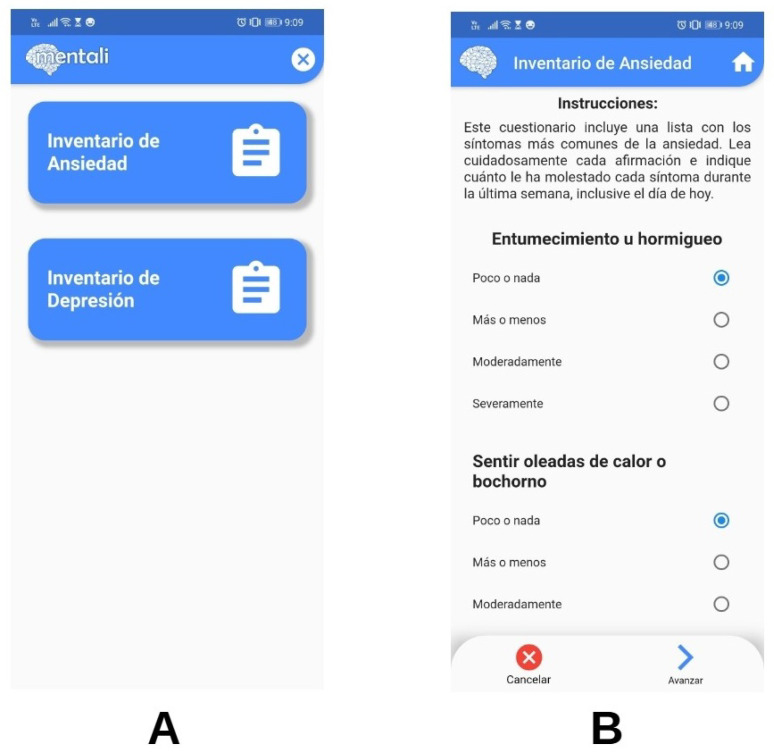
Select inventory (**A**) and anxiety inventory (**B**) screens.

The inventory module was also developed as part of the first iteration. This module allows the user to respond to the Beck Anxiety Inventory and the Beck Depression Inventory so that their mental health status can be evaluated and specialists enabled to obtain a primary screening. Screenshots of this module can be seen in Figure 9A,B.

**Figure 10:**
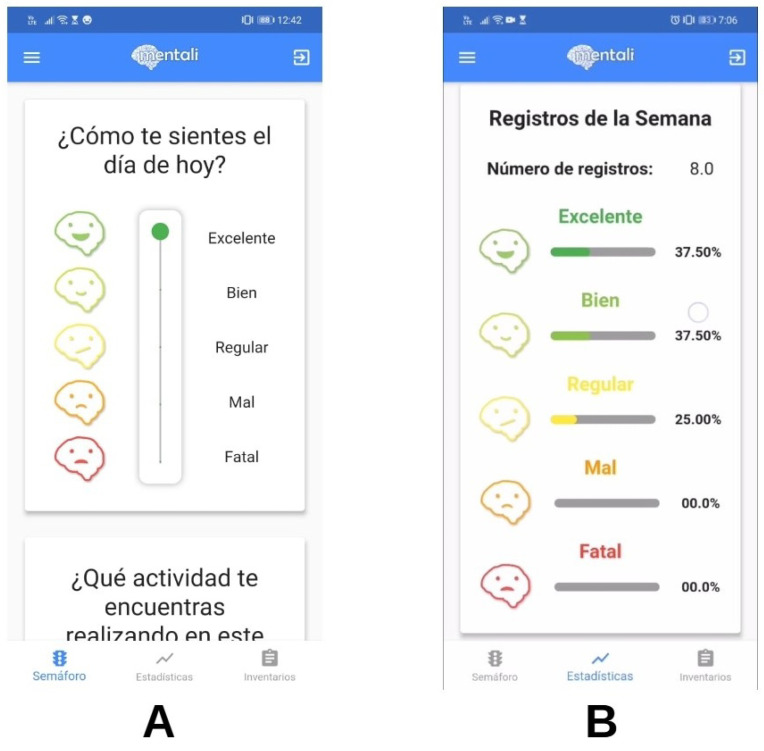
Mood log (**A**) and statistics (**B**) screens.

For the second iteration, user stories corresponding to the mood module were implemented. First, we developed a feature for users to record their mood on a scale from Terrible to Excellent, in addition to recording the activity they were performing at the time of registration (see Figure 10A).

As a second module, we implemented a feature in which users could view mood logs represented in the form of different types of charts. For instance, users could visualize how many records for each scale they had for the current week. They could also review the distribution of the current month’s records and display a pie chart with the records divided by scales for each day of the current week (see Figure 10B).

#### 3.1.3. Testing

As part of the verification activities, it was planned to test the mobile application at the end of each iteration, before delivering the incremental iteration to the customer. Unit tests were performed on the components considered essential for each of the user stories within each module. The integration tests carried out were performed to evaluate the correct functioning of each user history. The tests were conducted in rounds, where failed round tests entered a correction process and were run again in the next round until they were considered to be successful.

For the first iteration, user and inventory modules were tested. The first part of Table 1 shows the total number of unit tests performed in the first iteration. At the beginning, a total of 47 tests were planned and at the end of the iteration a total of 72 tests were completed. A total of 25 of these, representing 34.72%, failed; the remaining 47 tests, representing 65.28%, were successfully completed.

The second part of Table 1 shows the total number of integration tests performed during the first iteration. At the beginning, a total of 15 tests were planned; after completing the iteration, a total of 22 tests were performed. A total of seven of these, representing 31.81%, failed; the remaining 15 tests, representing 68.19%, were successfully completed.

In total, considering both the unit and integration tests, 62 tests were planned during the first iteration. During development, a total of 94 tests were carried out, of which 34.04% were failed tests and the remaining 65.96% were successful.

For the second iteration, tests were performed on the mood module. The first part of Table 2 shows the total number of unit tests performed in the second iteration. At the beginning, a total of 10 tests were planned; when developing the phase, a total of 23 tests were performed. A total of 13 of these, representing 60.86%, failed; the remaining 10 tests, representing 39.14%, were successfully completed.

The second part of Table 2 shows the total number of integration tests performed in the second iteration. At the beginning, a total of five tests were planned; when developing the phase a total of eight tests were performed. A total of three of these, representing 37.5% failed; the remaining five tests, representing 62.5%, were successfully completed.

In total, considering both the unit and integration tests, 15 tests were planned for the second iteration. When the phase was developed, a total of 31 tests were performed, of which 51.61% were failed tests and the remaining 48.38% were successful.

#### 3.1.4. Deployment

Currently, the Mentali mobile app is available in the top three mobile app stores: Play Store (https://play.google.com/store/apps/details?id=mx.com.mentali.mentali&hl=es_MX&gl=US, accessed on 12 March 2021), for Android devices; App Store (https://apps.apple.com/sv/app/mentali/id1555977677, accessed on 12 March 2021), for iOS devices and App Gallery (https://appgallery.huawei.com/#/app/C103622393, accessed on 12 March 2021) for Huawei devices.

### 3.2. Web Application

This section presents the results of the development, testing and deployment phases for the web application.

#### 3.2.1. Development

**Figure 11:**
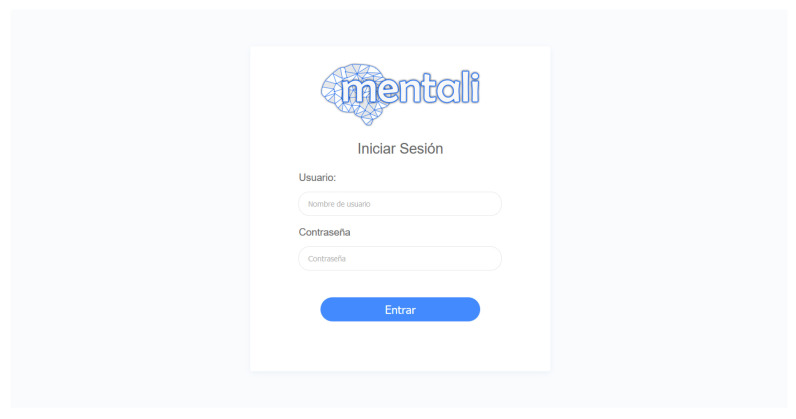
Login screen.

As a result of the web application development process, screenshots of the developed modules were displayed. During the first iteration, stories corresponding to the user module, i.e., the login and logout stories, were implemented. With these functionalities, the user can login to the system using a username and a password that are granted by the development team. This is because we decided that, in the initial iterations, the account creation functionality was not developed because the system was used by only a few specialists from the Molecular Medicine Laboratory. In Figure 11, the login page is shown.

**Figure 12:**
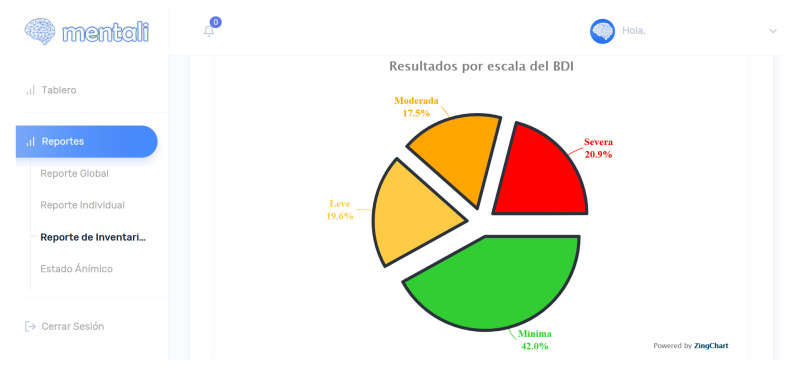
Inventories screen—pie chart.

For the second iteration, the user histories corresponding to the inventory module were implemented. With these functionalities implemented, the specialist can visualize the data for the results of the inventories in the form of linear and pastel graphs, as well as being able to generate reports. In Figure 12, the results of this iteration can be seen.

**Figure 13:**
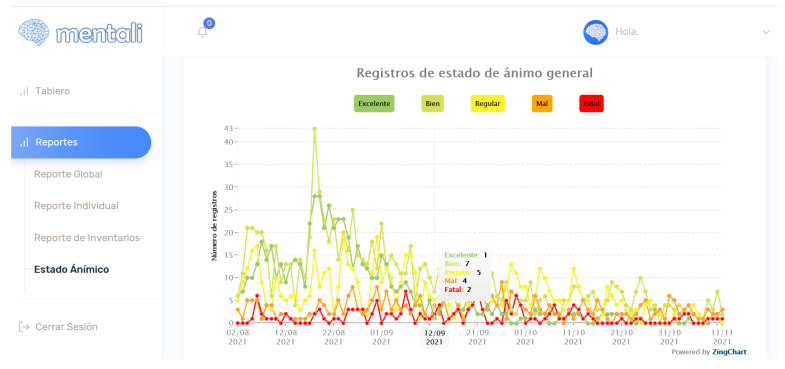
Mood screen—linear chart.

In the last iteration, user stories of the mood log module were implemented. The functionalities of this module enable the specialist to view mood log data in the form of a linear graph and a word cloud. In addition, the specialist can generate a report in Excel with the mood data. One of these features can be seen in Figure 13.

#### 3.2.2. Testing

In a similar way to the mobile application, within the verification activities, we planned to test the web application at the end of each iteration. Unit tests were performed on the components considered essential in each of the user stories within each module. The acceptance tests carried out were performed to evaluate the correct operation of each user history.

For the first iteration, user stories from the user’s module were tested. The first part of Table 3 shows the total number of unit tests performed in the first iteration. At the beginning, a total of 14 tests were planned; when developing the phase, a total of 22 tests were performed. A total of eight of them, representing 36.36%, failed; the remaining 14 tests, representing 63.64%, were successfully completed.

The second part of Table 3 shows the total number of acceptance tests performed during the first iteration. At the beginning, a total of five tests were planned; when developing the phase a total of six tests were performed. One of them, representing 16.66%, failed; the remaining five tests, representing 83.34%, were successfully completed.

In total, considering the unit and integration tests, 28 tests were performed during the first iteration, of which 22 were unit tests and six were acceptance tests, representing 78.57% and 21.43%, respectively.

For the second iteration, user histories of the inventories module were tested. In the first part of Table 4, the total number of unit tests performed on the module is shown. At the beginning, a total of 13 tests were planned; when developing the phase, a total of 25 tests were performed. A total of 12 of these, representing 48%, failed; the remaining 13 tests, representing 52%, were successfully completed.

The second part of Table 4 shows the total number of acceptance tests performed on the inventory module in the second iteration. At the beginning, a total of two tests were planned; when developing the phase, a total of five tests were performed. A total of three of them, representing 60%, failed; the remaining two tests, representing 40%, were successfully completed.

In total, considering the unit and acceptance tests, 15 tests were planned for the inventory module. When the phase was developed, a total of 30 tests were carried out, of which 50% were failed tests and the remaining 50% were successful.

For the third iteration, tests were performed on the mood module. The first part of Table 5 shows the total number of unit tests performed. At the beginning, a total of 13 tests were planned; when developing the phase, a total of 19 tests were performed. A total of six of them, representing 31.57%, failed; the remaining 13 tests, representing 68.43%, were successfully completed.

The second part of Table 5 shows the total number of integration tests performed on the mood module in the third iteration. At the beginning, a total of two tests were planned; when developing the phase, a total of three tests were performed. One of them, representing 33.33%, failed; the remaining two tests, representing 66.67%, were successfully completed.

In total, 22 tests were performed during the third iteration, of which 19 were unit tests and three were acceptance tests, representing 86.36% and 13.64%, respectively.

### 3.3. Implementation

In the period from August to December 2021, a pilot test of the use of the application was carried out in which students from eight groups of the first semester of the Academic Program of Human Medicine of the UAZ were invited to participate. A total of 201 students downloaded the app; 46 (22.9%) of these students were eliminated from the study because they did not interact with Mentali. Of the remaining 155 students, 100 were female and 55 were male, aged between 17 and 26 years. With registration of these users, it was possible to verify that the user stories corresponded to the creation of the user accounts and that the login worked correctly.

A total of 121 (78%) Mentali users interacted at least once with the Beck inventories module and 139 (89.6%) routinely interacted with the mood module. The students were asked to respond to the anxiety and depression inventories every 14 and 7 days as appropriate. A total of 83 users responded at least once to the depression inventory. The results of these records were as follows: minimal 29.7%, mild 15.7%, moderate 16.9% and, finally, severe 37.7%. According to these results, the scale with the highest percentage was severe, followed by the minimal scale.

A total of 93 users responded at least once to the anxiety inventory. The results of these records were as follows: minimal 26.9%, mild 10.5%, moderate 40.1% and, finally, severe 22.5%. In this case, the scale with the highest percentage was the moderate scale, which makes it necessary to pay attention to these results. The mobile application was responsible for processing the results of both inventories, and, according to the results, to classify them in the scales referred to above. With the results obtained, it was possible to validate the correct functioning of the user stories against the registration of the inventories.

Another activity that participants in the pilot study were asked to perform was to complete the mood section, where they could indicate how they felt according to the scale provided, as well as being able to indicate the current activity they were engaged in. A total of 139 users responded at least once to the mood record. Of a total of 2845 mood logs, the mood ’Good’ represented the highest percentage of responses with 35.149% of the logs; however, it is important to note that there were a considerable number of logs (502) with ’Bad’ and ’Fatal’ responses, which represented 17.6% of the logs.

To complement the pilot, the web application periodically performed analysis of the data generated and indicated if any of the users should be directed towards a specialist to be evaluated. Forty such alerts were received, of which 15 were identified based on the results of the anxiety inventory, nine based on the results of the mood log, 12 based on both the results of the anxiety and depression inventory, three based on the results of the anxiety inventory and the mood log, and one based on the results of the three components. The identification process started exactly 15 days after the implementation had commenced with the students of the first semester—this was due to the characteristics of the algorithm used. Following the alerts, all users were contacted to provide them with the necessary care. A total of 26 of those contacted accepted the offer of care and, for these, the psychological diagnoses and the need for care were corroborated by mental health specialists.

## 4. Discussion

The aim of this study was to develop a platform comprising a mobile and a web application (Mentali) to be used as an auxiliary tool for the detection of anxiety and depression in young adults through analysis of the results of validated inventories and an assessment of the user’s mood.

Prior to the development of the platform, a systematic review was conducted [11]. One of the main findings of this review was that the most common problems encountered with mobile applications were related to usability. For this reason, the articles selected in the literature review provided recommendations, such as user-centered design, usability tests, and consultation with health professionals to avoid many of the problems previously encountered with this type of application. As the main strategy to avoid usability problems for the developed platform, a participatory design workshop was implemented with potential users of the mobile application. This workshop enabled identification of users’ preferences and concerns regarding the use of a mobile application for assessment of mental health. We discovered that the frequency of users’ mobile phone use was high and that users were willing to use a mental health app because they were interested in their well-being to achieve better results in college. With these results, we were able to strengthen our proposal.

During the development of the Mentali platform, a number of very important observations were made. When developing a software system for use in different fields, it is important to consider specific needs. In this case, identification of the needs of users was vital in ensuring that the mobile application was attractive to users and encouraging its use. The feedback that was obtained after each iteration was developed enabled identification of possible changes and improvements to the application to meet the needs of users.

One of the main questions arising during the project was whether, using the platform developed, it was possible to identify students with conditions such as anxiety and depression, and, in addition, whether this could be carried out in a shorter time compared to procedures used previously. Based on the results obtained so far relating to implementation of the platform with first semester students of the academic program of human medicine, it can be concluded that the platform fulfilled the objective of identifying those students who may present with conditions such as anxiety and depression, using the Beck inventories. In addition, the results of the inventories do not require more than 30 seconds to be reviewed from the web application after the user has made a registration, confirming that the time taken to obtain inventory results was significantly reduced.

Another question identified at the beginning of this project concerned the most frequent factors that may trigger conditions such as anxiety, depression, stress and distress in students. With the help of the web application, managers are provided with a list of possible factors from which it is possible to generate strategies that can facilitate improvement in the environment of students, and, thereby, contribute to reducing the presence of these factors. The word cloud that was part of the state of mind report was the component that provided this list of factors, which were identified from the students’ mood log. According to the the currently available information, the most worrying factors at the moment for the students who used the mobile app were homework, classes and study. Some subjects studied on the degree in human medicine were mentioned frequently.

With the information obtained from the pilot study, it was possible to determine that the most frequent reported condition among the participating students was anxiety, representing 77.5% of the alerts received. This information is very important because, based on it, is possible to develop strategies that can contribute to reducing this condition in students. Fortunately, of the 40 students identified and contacted, 26 received support from a specialist. With the help of the platform, it was possible to identify vulnerable students and to channel them in a period of two to three weeks based on the response times for each of the inventories. The results confirm that the use of the platform can significantly reduce the time required to channel students towards a specialist compared to methods previously used within the human medicine department at UAZ.

Our results showed that 77.1% of the users who installed Mentali interacted with it. A considerable percentage of the participant sample (89.6%) interacted with the mood module. Among the participants who interacted with the mood module, for 40%, this was the only module that they used. The inventories were only used by 60% of the users. Some studies have investigated interest in using apps that help in managing stress in relation to the personality of users. For example, Ref. [18] showed that individuals with high neuroticism were more interested in using stress management apps that may benefit them, while individuals with low agreeableness scores tended to express lower interest in using mobile stress management apps. With the same concern, recognizing that stress can be expressed in a more hectic phone usage pattern than on normal days, in an interesting proposal, Vildjiounaite et al., developed a novel unsupervised stress detector, based on using a smartphone and using discrete hidden Markov models with maximum posterior marginal decisions for analysis of phone data. The feasibility of the proposed approach was confirmed in experiments using real-life data, which demonstrated accuracies comparable to those for fully supervised methods reported in other studies [19]. In addition to the demands and time involved in answering questionnaires with numerous items, together these results indicate the need to develop new anxiety and/or depression screening tests that are more attractive for young adults. Furthermore, future mobile interventions assessing coping strategies for stress and/or anxiety management should consider both unobtrusive strategies and user personality to improve user engagement and health support [18,19,20].

Finally, it is important to note that Mentali has also previously enabled coordination of the provision of specialized interventions, allowing administrators of the app to increase the proportion of patients who needed psychological care and subsequently received it by 30%, and the identification of several variables related to user’s mental health conditions according to stage of life [20]. In addition, Mentali has been found to be beneficial in determining the relationship between academic activities over time with effects on mood and mental health. Such information may be of value to academic authorities to create healthier academic environments [20].

## 5. Conclusions

By implementing the platform with medical students, it was possible to confirm that both applications achieved their main objectives. It was possible to channel students who were identified in the primary screening towards specialists, taking a maximum of two weeks based on the results of two inventories. The time taken to identify students needing to see a specialist was drastically reduced compared to traditional methods. In addition, most students were willing to meet with a specialist if this was recommended. Sometimes it is difficult to find a way to approach a specialist—with a platform like Mentali, this is made easier.

From the results obtained from the development stages of this project, the feasibility of developing mobile and web applications that enable primary screening with the aim of focusing resources on specific populations has been demonstrated. It is important to stress that the development of these applications must be supported by specialists in the field of study, as well as being scientifically validated, and respect previously established procedures. We consider it to be extremely important to continue research and development for software systems in the field of health to allow more people to benefit from these resources and to improve their quality of life.

## 6. Patents

The web application was registered with the Instituto Nacional del Derecho de Autor on 4 January 2022 (Registration number: 03-2021-121414575000-01). The registration of the mobile application was made on 10 January 2022 (Registration number: 03-2021-090711134000-01).

## Figures and Tables

**Table 1:** Table of tests performed during the first iteration.

Round	Successful Tests	Failed Tests	Total
**Unit Tests**			
1	26	21	47
2	18	3	21
3	2	1	3
4	1	0	1
Total	47	25	72
**Integration Tests**			
1	10	5	15
2	3	2	5
3	2	0	2
Total	15	7	22
**Total**	**62**	**32**	**94**

**Table 2:** Table of tests performed during the first iteration.

Round	Successful Tests	Failed Tests	Total
**Unit Tests**			
1	2	8	10
2	4	4	8
3	3	1	4
4	1	0	1
Total	10	13	23
**Integration Tests**			
1	4	1	5
2	0	1	1
3	0	1	1
4	1	0	1
Total	5	3	8
**Total**	**15**	**16**	**31**

**Table 3:** Table of tests performed during the first iteration.

Round	Successful Tests	Failed Tests	Total
**Unit Tests**			
1	8	6	14
2	4	2	6
3	2	0	2
Total	14	8	22
**Acceptance Tests**			
1	4	1	5
2	1	0	1
Total	5	1	6
**Total**	**19**	**9**	**28**

Successful tests were those that met the specified criteria in the user story. Failed tests were those that did not meet these criteria.

**Table 4:** Table of tests performed during the first iteration.

Round	Successful Tests	Failed Tests	Total
**Unit Tests**			
1	5	8	13
2	5	3	8
3	2	1	3
4	1	0	1
Total	13	12	25
**Acceptance Tests**			
1	0	2	2
2	1	1	2
3	1	0	1
Total	2	3	5
**Total**	**15**	**15**	**30**

Successful tests were those that met the specified criteria in the user story. Failed tests were those that did not meet these criteria.

**Table 5:** Table of tests performed during the first iteration.

Round	Successful Tests	Failed Tests	Total
**Unit Tests**			
1	8	5	13
2	4	1	5
3	1	0	1
Total	13	6	19
**Acceptance Tests**			
1	1	1	2
2	1	0	1
Total	2	1	3
**Total**	**15**	**7**	**22**

Successful tests were those that met the specified criteria in the user story. Failed tests were those that did not meet these criteria.

## Data Availability

All data supporting the reported results are included in the manuscript. Additional information regarding data that support the findings of this study will be available from the corresponding author [M.d.l.L.M.-F.] upon reasonable request.

## Abbreviations

The following abbreviations are used in this manuscript:

UAZ	Universidad Autónoma de Zacatecas

## References

[1] Organización Mundial de la Salud Depresión. https://www.who.int/es/news-room/fact-sheets/detail/depression.

[2] Organización Mundial de la Salud Salud Mental. https://www.who.int/topics/mental_health/es/.

[3] Medina-Mora M.E., Borges G., Muñoz C.L., Benjet C., Jaimes J.B., Bautista C.F., Velázquez J.V., Guiot E.R., Ruíz J.Z., Rodas L.C. (2003). Prevalencia de trastornos mentales y uso de servicios: Resultados de la Encuesta Nacional de Epidemiología Psiquiátrica en México. Salud Ment..

[4] Belló M., Puentes-Rosas E., Medina-Mora M.E., Lozano R. (2005). Prevalencia y diagnóstico de depresiónen población adulta en México. Salud Publica Mex..

[5] Wang C., Pan R., Wan X., Tan Y., Xu L., Ho C.S., Ho R.C. (2020). Immediate psychological responses and associated factors during the initial stage of the 2019 coronavirus disease (COVID-19) epidemic among the general population in China. Int. J. Environ. Res. Public Health.

[6] Gutiérrez Rodas J., Montoya Vélez L., Toro Isaza B., Brión Zapata M., Rosas Restrepo E., Salazar Quintero L. (2010). Depresión en estudiantes universitarios y su asociación con el estrés académico. CES Med..

[7] Soria Trujano R., Morales Pérez A.K., Ávila Ramos E. (2015). Depresión y problemas de salud en estudiantes universitarios de la carrera de Medicina. Diferencias de género. Altern. Psicol..

[8] Beck A.T., Steer R. (1988). Beck anxiety inventory (BAI). Überblick über Reliabilitäts-und Validitätsbefunde von klinischen und außerklinischen Selbst-und Fremdbeurteilungsverfahren. J. Consult. Clin. Psychol..

[9] Beck A.T., Steer R.A., Brown G.K. (1987). Beck Depression Inventory.

[10] Donker T., Katherine P., Proudfoot J., Clarke J., Birch M.R., Christensen H. (2013). Smartphones for Smarter Delivery of Mental Health Programs A Systematic Review Donker Journal of Medical Internet Research. J. Med. Internet Res..

[11] Solís-Galván J.A., Vázquez-Reyes S., Martínez-Fierro M., Velasco-Elizondo P., Garza-Veloz I., Caldera-Villalobos C. (2020). Towards Development of a Mobile Application to Evaluate Mental Health: Systematic Literature Review. Proceedings of the International Conference on Software Process Improvement.

[12] Schwaber K., Sutherland J. (2011). The Scrum Guide: The Definitive Guide to Scrum: The Rules of the Game. https://www.scrum.org/resources/scrum-guide.

[13] Team M., Safari A.O.M.C. (2009). Microsoft® Application Architecture Guide.

[14] Dart Programming Language|Dart. https://dart.dev/.

[15] Flutter—Build Apps for Any Screen. https://flutter.dev/.

[16] The Web Framework for Perfectionists with Deadlines|Django. https://www.djangoproject.com/.

[17] Welcome to Python.org. https://www.python.org/.

[18] Ervasti M., Kallio J., Määttänen I., Mäntyjärvi J., Jokela M. (2019). Influence of Personality and Differences in Stress Processing Among Finnish Students on Interest to Use a Mobile Stress Management App: Survey Study. JMIR Ment. Health.

[19] Vildjiounaite E., Kallio J., Kyllönen V., Nieminen M., Määttänen I., Lindholm M., Mäntyjärvi J., Gimel’farb G. (2018). Unobtrusive stress detection on the basis of smartphone usage data. Pers. Ubiquitous Comput..

[20] Martinez-Fierro M.L., Ayala-Haro A.E., Pinedo-Hurtado M.E., Solis-Galvan J.A., Garza-Veloz I., Velazquez-Lopez Z.Y., Camacho-Martinez A.G., Avila-Carrasco L., Vazquez-Reyes S., Velasco-Elizondo P. (2022). Usefulness of a Mobile Application (Mentali) for Anxiety and Depression Screening in Medical Students and Description of the Associated Triggering Factors. Brain Sci..

